# Bacterial diversity and resistome analysis of drinking water stored in cisterns from two First Nations communities in Manitoba, Canada

**DOI:** 10.1128/spectrum.03141-23

**Published:** 2024-02-02

**Authors:** Anita Murdock, Sabrin Bashar, Dawn White, Miguel Uyaguari-Diaz, Annemieke Farenhorst, Ayush Kumar

**Affiliations:** 1 Departments of Microbiology, University of Manitoba, Winnipeg, Canada; 2 Soil Science, University of Manitoba, Winnipeg, Canada; Dominican University New York, Orangeburg, New York, USA

**Keywords:** bacteria, antibiotic resistance genes, water treatment, water storage

## Abstract

The microbiological content of water is an ongoing concern in First Nations communities in Canada. Many communities lack water treatment plants and continue to be under drinking water advisories. However, lack of access to treatment plants is only a part of the problem as poor water distribution systems also contribute to the failure to provide safe drinking water. Here, we studied the microbial diversity and antibiotic resistome from water stored in cisterns from two First Nations communities in Manitoba, Canada. We found that the cistern water contained a high number of bacteria and showed the presence of diverse antimicrobial resistance genes. Interestingly, the bacterial diversity and antimicrobial resistance genes varied considerably from that of the untreated source water, indicating that the origin of contamination in the cistern water came from within the treatment plant or along the delivery route to the homes. Our study highlights the importance of proper maintenance of the water distribution system in addition to access to water treatment facilities to ensure a supply of safe water to First Nations communities in Canada.

**IMPORTANCE:**

The work described addresses a critical issue in First Nations communities in Canada—the microbiological content of water. Many of these communities lack access to water treatment plants and frequently experience drinking water advisories. This study focused on the microbial diversity and antibiotic resistome in water stored in cisterns within two First Nations communities in Manitoba, Canada. These findings reveal that cistern water, a common source of drinking water in these communities, contains a high number of bacteria and a wide range of antimicrobial resistance genes. This highlights a serious health risk as exposure to such water can lead to the spread of drug-resistant infections, posing a threat to the well-being of the residents.

## INTRODUCTION

Poor water quality within First Nations drinking water distribution systems is a well-known human rights concern that remains unresolved in Canada (1, 2). The most recent national assessment examining 740 of the 761 First Nations drinking water distribution systems classified 29% of the systems at high risk of negatively affecting drinking water quality and consequently the quality of life for community members (3). In the Province of Manitoba, Canada, surface water is the source for 50% of the drinking water distribution systems in First Nations communities (3), which has a greater risk than groundwater for chemical and bacteriological contamination by anthropogenic activities and wildlife. Additionally, 31% of the homes in First Nations communities in Manitoba require water delivery by truck for storage in cisterns (3). The quality of this stored water can deteriorate rapidly due to bacterial contamination as documented previously (4–6); so, even if the quality of the water is acceptable upon delivery, there is a need to monitor and maintain that quality during storage.

The global trend to overuse antibiotics in vital sectors (i.e., healthcare, agriculture, and veterinary medicine) has facilitated the development of antimicrobial resistance across many bacterial species. For example, carbapenem-resistant *Enterobacterales* (CRE) and extended spectrum β-lactamase (ESBL)-producing *Enterobacterales* cause an alarming number of healthcare-associated infections that are resistant to antibiotic treatment. An estimated 13,100 and 197,400 cases of CRE- and ESBL-producing *Enterobacterales*, respectively, occurred in the USA in 2017 (7). Carbapenem-resistant *Acinetobacter* was responsible for 8,500 hospitalizations in 2017; vancomycin-resistant enterococci caused 54,500 infections (7). In Canada, it is estimated that one out of nineteen deaths in 2018 was caused by antibiotic-resistant infections (8) and the 2021 surveillance report indicated increased resistance rates for three of the four monitored priority microorganisms (9). Neither report differentiates the data between indigenous and non-indigenous populations (information that is greatly lacking), but it is known that community-acquired methicillin-resistant *Staphylococcus aureus* affects First Nations people at a significantly higher rate than the remainder of the population (10, 11).

Microorganisms have always had the ability to acquire natural antibiotic-resistant determinants (12). It is now, since the introduction of manufactured antibiotics and their widespread use and subsequent flow into the environment, that antibiotics and antibiotic resistance genes (ARGs) are found more abundantly in various environmental niches (13–15). Constant exposure to antibiotics has forced bacteria to acquire ARGs—either through mobile genetic elements like plasmids or through the uptake of extracellular DNA—for their survival. Ultimately, this has led to the sharing of ARGs between bacteria (horizontal transfer) or with daughter cells (vertical transfer) and the consequential appearance of extensive antimicrobial resistance. The presence of ARGs in the environment, especially aquatic settings (including water treatment plants) (15–17), has led to them being classified as “emerging contaminants” as their presence represents a threat to increased development of new resistant strains of bacteria (18).

Bacterial contamination of drinking water is commonly found in the tap water of homes in First Nations communities due to water treatment insufficiencies or prolonged storage in underground cisterns (4–6). Given that one in three First Nations homes in Manitoba relies on cisterns as the sole source of potable water, it is critical to identify and quantify ARGs in this water supply to provide preliminary information about the exposure of a large population to ARGs as a result of household water. In a previous study of First Nations source and drinking water samples, we specifically aimed to identify three ARGs [*ampC*, *tet*(A), and *mecA*] using quantitative PCR and screened them for multiple β-lactamase and carbapenemase genes (5). In this work, using shotgun metagenomics, we studied the microbial communities and all known and suspected ARGs in cistern-held water from two First Nations communities with the goal to better understand the potential health risks associated with this common water storage method.

## MATERIAL AND METHODS

### Water samples

Water samples were collected from two First Nations reserves in Manitoba, designated as community B and community D (Table 1). Two source water samples were collected from the source water treatment plant (SWTP) prior to treatment (untreated) in each community (SWTP B and SWTP D). One post-treated water sample was collected from the kitchen tap of a home with an underground concrete cistern located in community B (Cistern B) and from a home with an underground fiberglass cistern located in community D (Cistern D). In addition, a water sample was collected from the Red River (RR) in the City of Winnipeg to serve as a reference for water heavily impacted by anthropogenic activities, including the release of wastewater treatment plant effluent and snowmelt runoff from agricultural land.

**TABLE 1:** Description of the water samples used in this study

Sample	Date	Community	Designation	Sample description
Red River	(September 2019)	Winnipeg	RR	Direct grab sample of surface water providing an untreated, anthropogenically impacted reference sample.
Source water treatment plant	October 2018	B and D	SWTP B and SWTP D	Source lake water taken at the water treatment plant drawn from a diversion pipe prior to treatment.
Concrete cistern	(August 2018)	B	Cistern B	Concrete constructed cistern housed underground external to the home. Intermittently filled by a water delivery truck. Exclusive to community B.
Fiberglass cistern	October 2018	D	Cistern D	Fiberglass constructed cistern housed under the home. Intermittently filled by a water delivery truck. Exclusive to community D.

### Sample collection

Sampling bottles were prepared at the University of Manitoba according to standard methods for sample bottle pre-treatment (17). Briefly, 1 L glass sampling bottles were sterilized using an autoclave at 121°C for 1 hour and opened only immediately prior to sampling. Samples were collected, preserved, and stored using recommended methods (17). A grab sample from the Red River was taken from the surface of the water; a sampling bottle was dipped until filled then sealed. Water samples from the treatment plants (SWTP) and the cisterns of community homes were taken from taps: any existing filters on the taps were removed and the surface of the tap was cleaned with a 90% ethanol wipe. Cold water was run for 3 minutes before filling the sampling bottles to ensure that the sample did not contain stagnant water from within the plumbing. These water sampling bottles were filled to 900 mL and 1 mL of a sterile 3% sodium thiosulfate solution was added to neutralize any residual chlorine and prevent bactericidal activity during transport to the laboratory. All samples were stored in coolers with ice packs during collection in the communities then at 4°C upon arrival on the same day at the University of Manitoba. Samples were processed <24 hours after collection.

### DNA extraction and metagenomic sequencing

Up to 700 mL of each water sample (one sample each from the Red River, Cistern B, and Cistern D; two samples from each community’s water source) was filtered through a 0.22-µm, gridded, 47-mm sterile polyether sulfone membrane filter (Pall Corporation, Mississauga, ON, Canada) to capture all intact bacteria and extracellular DNA. The filters were stored at −20°C until further processing. DNA was extracted from the filters using the DNeasy PowerWater Kit (Qiagen, Germantown, MD, USA) according to the manufacturer’s instructions. Concentration and purity of the purified DNA were determined using a Nanodrop 2000 spectrophotometer (Thermo Scientific, Waltham, MA, USA). Shotgun metagenomic sequencing was carried out using an Illumina MiSeq with paired end reads (250 bp) by Génome Québec (Montreal, Québec, Canada). Sequence data are publicly available at the National Center for Biotechnology Information, BioProject number PRJNA1005677.

### Data processing and visualization

Fastq sequence data files were uploaded to MetaStorm (19) for taxonomic and functional annotation using the read matching-based pipeline. Reads were trimmed and assessed for quality using Trimmomatic (20); Bowtie (21) was used for read alignment to bacterial reference genomes. Greengenes (22) was used to identify and annotate 16S rRNA gene sequences ahead of 16S rRNA normalization using the functional annotation pipeline in MetaStorm. The Comprehensive Antibiotic Resistance Database (CARD) v 3.0.2 (23) was used for ARG annotation against the sequences, leading to the calculation of ARG per copy of 16S rRNA. Data from Greengenes and CARD were exported into R (24) where RStudio (25) was applied to generate figures using *ggplot2* (26). Principal coordinate analysis (PCoA) was calculated using microbial order composition and antibiotic class resistance and processed using the R packages *vegan* and *stats* (24); the Bray-Curtis dissimilarity index was calculated to show metric multidimensional scaling.

## RESULTS

### Resistome profile

**Fig 1:**
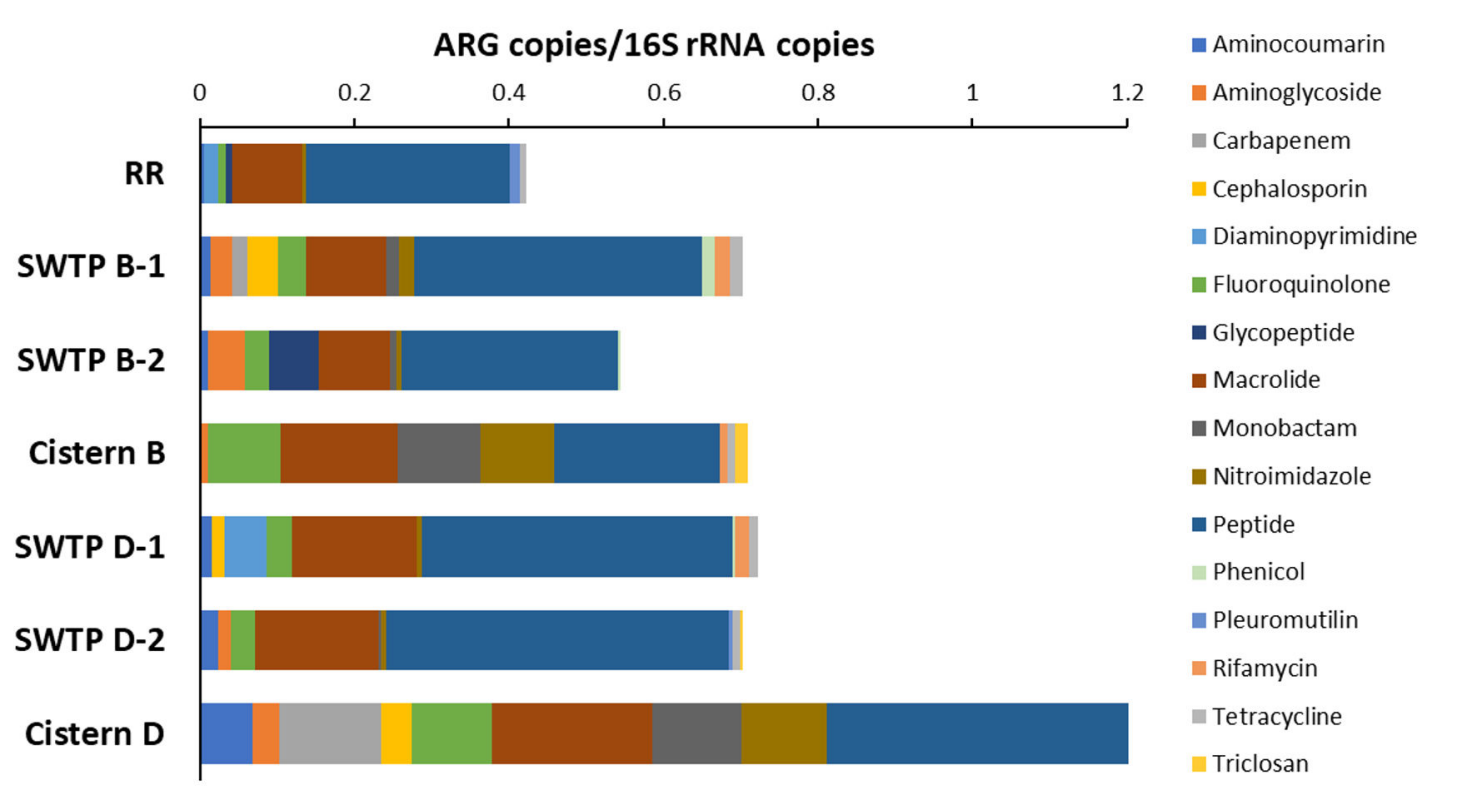
Determining the absolute resistome of two First Nations community water samples using the ratio of antibiotic resistance genes (ARGs) to 16S rRNA copies. Site and sample names are abbreviated as follows: Winnipeg Red River, RR; untreated source water from community B, SWTP B-1, and SWTP B-2; concrete cistern from community B, Cistern B; untreated source water from community D, SWTP D-1 and SWTP D-2; fiberglass cistern from community D, Cistern D.

**Fig 2:**
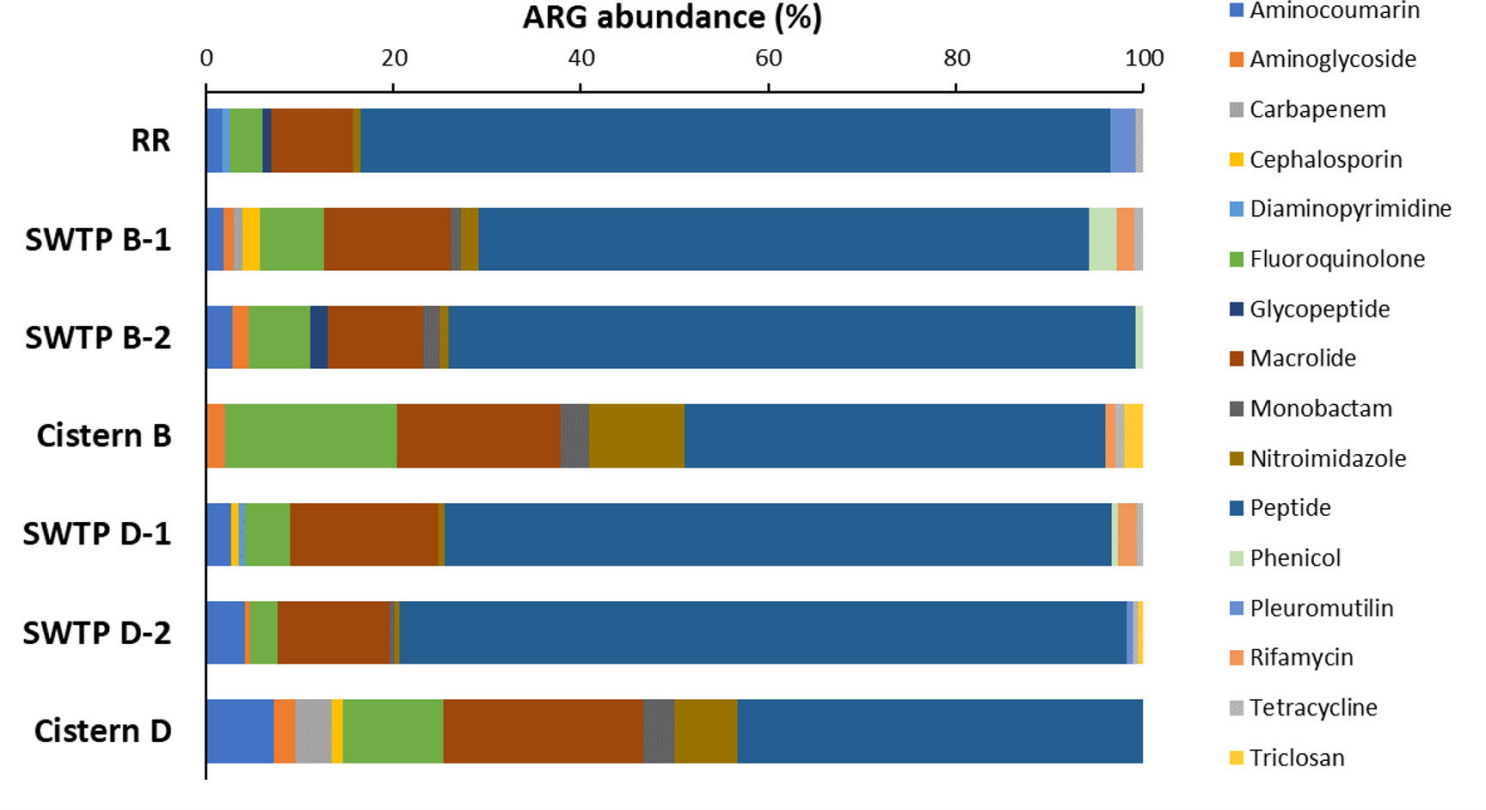
Determining the relative resistome of two First Nations community water samples using the percent abundance of antibiotic resistance genes (ARGs). Site and sample names are abbreviated as follows: Winnipeg Red River, RR; untreated source water from community B, SWTP B-1 and SWTP B-2; concrete cistern from community B, Cistern B; untreated source water from community D, SWTP D-1 and SWTP D-2; fiberglass cistern from community D, Cistern D.

The total resistome profile of samples taken from an SWTP and a home was established at each of the two communities using the absolute (Fig. 1) and relative (Fig. 2) ARG abundances expressed by resistance to antibiotic class. Overall, a total of 16 different antibiotic classes were observed. The top three most abundant classes were peptides, macrolides, and fluoroquinolones that were present in every sample (Fig. 1). This corresponded to an overall relative ARG abundance average of 61% for peptide resistance, 14% for macrolides resistance, and 10% for fluoroquinolone resistance in community B water samples and an abundance of 63% for peptide resistance, 16% for macrolides resistance, and 6% for fluoroquinolone resistance in community D water samples (Fig. 2). Antibiotic efflux pumps were the most prominent genes in the resistomes: of the 73 unique ARGs, 48 were related to various superfamilies of efflux pumps including the ATP-binding cassette, resistance nodulation division, and major facilitator efflux pump systems (Table 2).

**TABLE 2:** Unique resistance genes identified in resistome by sample^*a*^

Antibiotic class	RR	SWTP B-1	SWTP B-2	Cistern B	SWTP D-1	SWTP D-2	Cistern D
Aminocoumarin		*mdtB*	*mdtB*			*mdtB*	*mdtB*
	*mdtC*					*mdtC*	*mdtC*
		*novA*					
Aminoglycoside			*aac(3')-Ia*				
			*aac(6')-Iia*				
		*aac(6')-Iic*					
							*aph(3')-Ia*
				*acrD*			*acrD*
							*smeB*
						*smeR*	
Carbapenem		*FEZ-1*					
							*rm3*
							*SPG-1*
Cephalosporin							*OXA-21*
		*OXA-119*			*OXA-119*		
							*OXA-205*
Diaminopyrimidine	dfrA14						
					*dfrB6*		
Fluoroquinolone				*acrB*	*acrB*		*acrB*
	*acrF*		*acrF*				*acrF*
	*adeF*			*adeF*		*adeF*	*adeF*
		*ceoB*					*ceoB*
			*emrA*				
						*mexE*	
	*mexF*	*mexF*	*mexF*	*mexF*	*mexF*	*mexF*	*mexF*
	*mexI*	*mexI*		*mexI*	*mexI*		*mexI*
					*oprN*	*oprN*	*oprN*
							*oqxB*
Glycopeptide			*BRP(MBL*)				
	*vanSO*						
Macrolide		*axyY*				*axyY*	*axyY*
				*CRP*		*CRP*	
			*efrB*				
					*macB*	*macB*	
		*mexB*			*mexB*		*mexB*
					*mexC*		
							*mexD*
	*mexK*	*mexK*	*mexK*		*mexK*	*mexK*	*mexK*
	*mexQ*		*mexQ*	*mexQ*			*mexQ*
		*mexW*	*mexW*		*mexW*	*mexW*	*mexW*
				*mexY*			
	*mtrA*	*mtrA*	*mtrA*	*mtrA*	*mtrA*	*mtrA*	
			*muxB*	*muxB*	*muxB*	*muxB*	*muxB*
		*muxC*		*muxC*	*muxC*	*muxC*	*muxC*
							*oleB*
	*oleC*	*oleC*	*oleC*		*oleC*	*oleC*	
				*ompB*			
				*oprM*			*oprM*
				*smeD*			
		*smeE*		*smeE*			*smeE*
Monobactam				*golS*			*golS*
			*mdsB*			*mdsB*	*mdsB*
		*PER-2*					
							*TEM-126*
Nitroimidazole	*msbA*	*msbA*	*msbA*	*msbA*	*msbA*	*msbA*	*msbA*
Peptide		*arnA*			*arnA*	*arnA*	
	*bacA*				*bacA*		*bacA*
	*bcrA*	*bcrA*	*bcrA*				*bcrA*
				*MCR-5*			
		*rosA*	*rosA*		*rosA*		
	*rosB*	*rosB*					*rosB*
	*rpoB2*	*rpoB2*	*rpoB2*	*rpoB2*	*rpoB2*	*rpoB2*	*rpoB2*
	*ugd*	*ugd*				*ugd*	*ugd*
Phenicol		*mexN*	*mexN*		*mexN*		
Pleuromutilin	*taeA*					*taeA*	
Rifamycin		*efpA*		*efpA*	*efpA*		
					*rphA*		
					*rphB*		
Tetracycline				*otr(A*)			
					*otrC*	*otrC*	
	*tetA(48*)	*tetA(48*)					
Triclosan				*ompH*			
				*triC*		*triC*	

^*a*^ Blank cells represent absence of the gene.

Resistance genes identified from untreated water samples displayed similar and less variable total resistomes than those from cistern-held water samples. The Red River sample had the least diverse antibiotic classes, with resistance to the peptide and macrolide classes comprising 88% of ARG abundance (Fig. 2). The untreated source water samples as well as the Red River samples showed the presence of approximately 0.4–0.7 ARG copies/16S rRNA copies in total (Fig. 1). Similarly, untreated source waters showed minor differences in total resistome composition and relative ARG abundance within duplicates. Each duplicate untreated source water sample in community B (SWTP B-1 and SWTP B-2) shared resistance against aminocoumarin, aminoglycoside, fluoroquinolone, macrolide, monobactam, nitroimidazole, peptide, and phenicol; however, SWTP B-2 did not contain carbapenem, cephalosporin, rifamycin, or tetracycline resistance as seen in SWTP B-1. Glycopeptide resistance, found at 0.06 and 0.008 ARG copies/16S rRNA copies respectively, was only found in the SWTP B-2 and Red River samples (Fig. 1 and 2). Community D samples, SWTP D-1 and SWTP D-2, both shared cephalosporin, diaminopyrimidine, phenicol, and rifamycin resistance; SWTP D-2 contained aminoglycoside, monobactam, pleuromutilin, and triclosan resistance (Fig. 1).

Resistome analysis showed that the absolute abundance in post-treated water from the concrete cistern in community B (Cistern B) was 0.7 ARG copies/16S rRNA copies; the fiberglass cistern in community D (Cistern D) had a value of 1.2 ARG copies/16S rRNA copies (Fig. 1). In the concrete cistern, ARGs indicated resistance to aminoglycoside, fluoroquinolone, macrolide, monobactam, nitroimidazole, peptides, rifamycin, tetracycline, and triclosan (Fig. 1). Even though triclosan resistance comprised ~2% of the ARG abundance from the cistern sample, it was not detected in the other samples from community B (SWTP B-1 and SWTP B-2). The ARGs found to be higher in the concrete cistern (Cistern B) compared to the source water (SWTP B-1 and SWTP B-2) were for fluoroquinolone (18% versus 6%), monobactam (3% versus 1–2%), and nitroimidazole (10% versus 1%–2%) (Fig. 2). In community D, ARGs in the fiberglass cistern (Cistern D) indicated resistance to aminocoumarin, aminoglycoside, carbapenem, cephalosporin, fluoroquinolone, macrolide, monobactam, nitroimidazole, and peptides. Comparing with the relative ARG abundance observed in the source water of community D, the fiberglass cistern did not show detectable triclosan (0.5% in SWTP D-2), tetracycline (1% SWTP D-1 and SWTP D-2), rifamycin (2% in SWTP D-1), pleuromutilin (1% in SWTP D-2), and phenicol (0.5% in SWTP D-1) (Fig. 2).

The primary resistance genes identified were components of efflux pump machinery, and common resistance genes included aminoglycoside acetyltransferase enzymes and rifampin phosphotransferases (Table 2). A few other ARGs were present but relatively less abundant, for example, *OXA-119* (cephalosporin) and *tetA(48*) (tetracycline) were found in SWTP samples; *OXA-21* and *OXA-205* (cephalosporin), *MCR-5* (peptide), *SPG-1* (carbapenem), and *TEM-126* (monobactam) were detected in cistern samples.

### Microbial community profile

**Fig 3:**
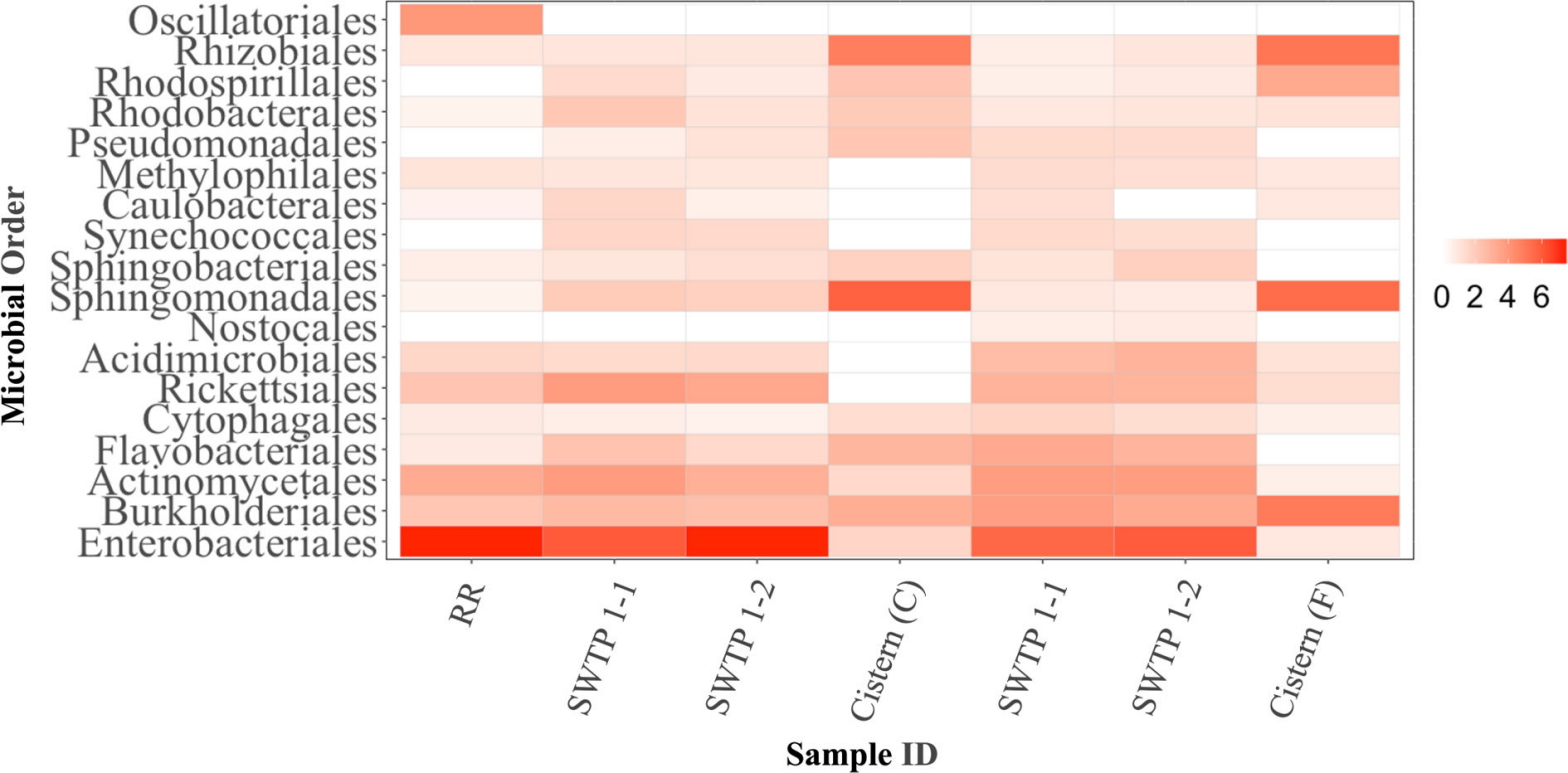
Determining the microbial community profile of two First Nations community water samples via the relative abundance of order-level bacteria normalized by 16S rRNA. The scale bar represents normalized abundance. Site and sample names are abbreviated as follows: Winnipeg Red River, RR; untreated source water from community B, SWTP B-1 and SWTP B-2; concrete cistern from community B, Cistern B; untreated source water from Ccmmunity D, SWTP D-1 and SWTP D-2; fiberglass cistern from community D, Cistern D.

The microbial composition of untreated water samples was compared with the microbial presence in cistern samples—water that had been treated, transported, and stored (Fig. 3). The microbial community profile across all water samples identified 18 different orders of bacteria (Fig. 3). Overall, the microbial community at the taxonomy level of order did not deviate between the duplicates of the untreated water samples collected from the diversion pipe at the water treatment plant (i.e., SWTP B-1 versus SWTP B-2) in either community. In community B, prominent bacterial orders included *Enterobacterales*, *Actinomycetales*, and *Rickettsiales*; in community D, the dominant bacterial orders were *Enterobacterales*, *Actinomycetales*, and *Burkholderiales*.

The microbial composition of each of the cistern-held water samples (Cistern B and Cistern D) was markedly different than that of the corresponding untreated water samples (SWTP) but, interestingly, similar to each other despite the difference in cistern composition and geographical location of the communities (Fig. 3). In both communities, on average, *Enterobacterales* was more abundant in the SWTP samples than the cistern water samples. Conversely, *Rhodobacterales* and *Rhodospirillales* were more dominant in the cistern water samples, on average, than the SWTP samples.

### ARG and microbial community analysis

**Fig 4:**
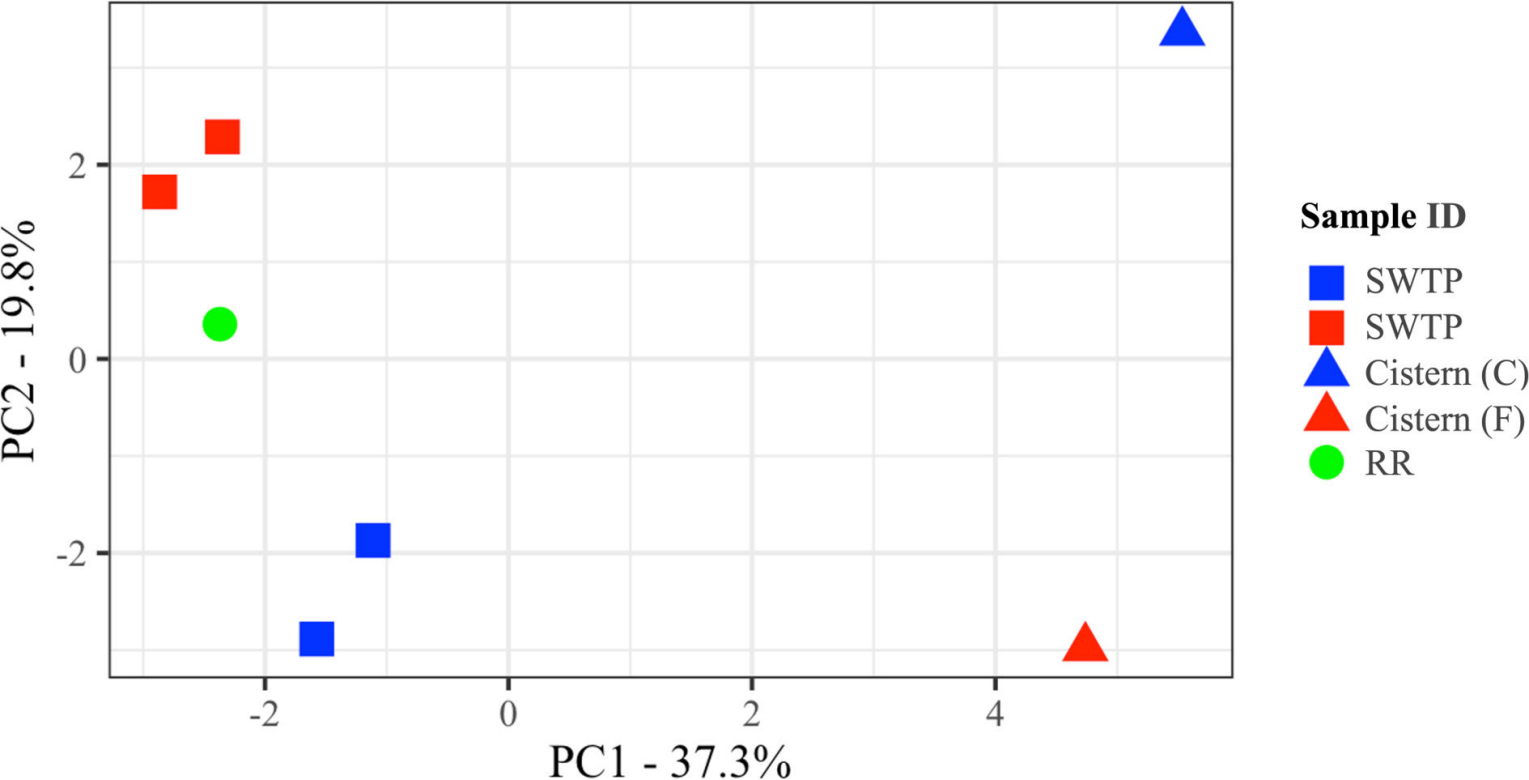
Principal coordinate analysis showing dissimilar relation of untreated source water (raw and untreated water) and post-treated cistern water samples from two First Nations communities. Site and sample names are abbreviated as follows: Winnipeg Red River, RR; untreated source water from community B, SWTP B-1 and SWTP B-2; concrete cistern from community B, Cistern B; untreated source water from community D, SWTP D-1 and SWTP D-2; fiberglass cistern from community D, Cistern D.

Having identified the total resistome and microbial communities in each water sample, the presence of persistent ARGs and microbial orders was used to evaluate the degree of dissimilarity and, therefore, contamination of cisterns by source water (Fig. 4). The PCoA plot accounted for 78.7% of the variation in the samples using PC1 (66.7%) and PC2 (12%). The concrete (Cistern B) and fiberglass (Cistern D) cistern water samples showed some diversity relative to each other and were exceptionally distinct from their matched community SWTP water samples and the reference river water sample (RR).

## DISCUSSION

In previous work, we showed that cistern water in First Nations reserves in the Province of Manitoba can contain high levels of fecal bacteria and ARGs even though these communities have fully functional water treatment plants (4–6). In a study in Nunavut, Canada, the detection of *Escherichia coli* in a cistern water sample collected from a household was attributed to the time delay between the cistern running dry and being refilled (27) suggesting that cisterns can pose a greater risk to the community if they are not refreshed and cleaned on a regular basis. The placement of cisterns can also impact their rate of contamination. For example, a study that included three First Nations reserves in Manitoba demonstrated that above-ground polyethylene cisterns provided safer drinking water than below-ground concrete or fiberglass cisterns (28). Common ARGs have frequently been reported in drinking water (5, 6, 29), but little is known about the greater antibiotic resistome. In this study, we expanded upon previous work on fecal indicator organisms and common, targeted resistance genes by determining the complete antibiotic resistome and microbial community profile of cistern-held water and its source water. This information will help us better understand where the contamination is coming from and point to potential solutions.

### Untreated waters

Thirty-two unique ARGs were identified in the untreated water samples (SWTP) from community B, and 40 were identified in SWTP samples from community D. Using the CARD database (23), five were recognized as clinically relevant—ARGs that play a mechanistic role in antibiotic resistance to known, employed antibiotics (30). The SWTP samples from community B contained four relevant ARGs, specifically *OXA-119* (cephalosporin resistance), *FEZ-1* (carbapenem resistance), *PER-2* (monobactam resistance), and *tetA(48*) (tetracycline resistance); only one, *OXA-119*, was found in the SWTP samples from community D (Table 2) (23).

The resistome profile of each community’s SWTP samples showed higher ARG abundance (measure against 16S rRNA copy number) (Fig. 1) and greater ARG diversity (Fig. 2) compared to the reference water sample from the Red River (RR), which did not contain carbapenem-, cephalosporin-, or monobactam-associated resistance genes as the SWTP samples did (Table 1). This suggests that the water distribution system within each First Nations community that we studied is a point where ARGs can be introduced or enriched. Other studies have had similar observations. For example, as study from Brazil showed an increase in the abundance of pathogens between the raw water source (5%) and the final tap water source (18%) (31). However, this study also showed that the ARG composition was identical when comparing raw and tap water samples: β-lactams (19%), macrolides (13%), fluoroquinolones (9%), and glycopeptides (3%) were most prevalent, and there was a greater ARG diversity in the raw water sample versus the tap water sample (31). Similarly, an increase in pathogen detection was reported after water treatment in two rural Saskatchewan, Canada, villages (32). Taken together, water distribution systems may be an important source of pathogen contamination that then leads to issues where the water is delivered.

### Cistern water

The concrete cistern of community B harbored 24 ARGs but only one clinically significant gene, *MCR-5* (colistin resistance). Thirty-two ARGs were identified in the fiberglass cistern of community D, four of which are clinically relevant: *SPG-1* (carbapenem resistance), *OXA-205* and *OXA-21* (cephalosporin resistance), and *TEM-126* (monobactam resistance) (Table 2). The absolute ARG abundance within each cistern water sample was equivalent or greater than the ARG abundance of its matched SWTP water sample (Fig. 1) suggesting that water treatment might not be removing the source of the ARGs originating from the raw water, and/or ARGs are being re-introduced during water distribution. Previous studies have shown that the level of normalized ARGs/bacterial count in concrete and fiberglass cisterns was comparable to the level determined in anthropogenically impacted raw water (33) and that ARG levels from cisterns were considerably lower than that quantified in wastewater effluents (34). This study cannot confirm the sources of the observed high abundance of ARGs in cistern water in the two communities, but previous studies have indicated that contamination may be due to inadequate chlorine residuals, external contamination introduced during maintenance or repair, contamination by sewage or heavy rainfall (35), or human activity in and around the cistern (pets and children) (36).

β-Lactamase ARGs appeared in both pre- and post-treated (cistern) water samples. OXA β-lactamases are categorized by molecular structure into four groups, A through D, according to the Ambler class system (37). Class D enzymes are predominantly plasmid-encoded β-lactamases in Gram-negative bacteria that are able to hydrolyze oxacillin and penicillin (38); they were the only class identified in our water samples. We found *OXA-119* in samples SWTP B-1 and SWTP D-1 (prior to treatment) and *OXA-21* and *OXA-205* in Cistern D (post-treatment). The presence of β-lactamase genes in drinking water has been reported previously. For example, one study reported β-lactam resistance gene abundance of 34% in treated water samples yet to be distributed and of 76% in tap water samples (39). Another study showed that OXA-related genes comprised 19.6% of the total ARGs detected in raw, disinfected, and tap waters (31). Each of the *bla_oxa_* genes identified in our study has reported to be present on mobile genetic elements. *OXA-119* (with 96% homology to *OXA-205*) was isolated from an integron in *Burkholderia cepacia* (40); *OXA-205* was isolated from an imipenem-resistant isolate of *Pseudomonas aeruginosa* containing the class 1 integron *In671* (40), and *OXA-21* was the first example of an *OXA*-related gene occurring outside of *P. aeruginosa*, discovered during a hospital respiratory disease outbreak caused by *Acinetobacter baumannii* (41). The presence of *bla_oxa_* genes, potentially in mobile genetic elements, poses an additional risk of transfer of these genes to other organisms present in the water samples.

Analysis of the 16S rRNA sequence data from the metagenomes revealed the presence of bacteria from multiple orders across all of our water samples. While *Enterobacterales* was consistently abundant in SWTP water samples, this order was nearly absent in each of the cistern water samples (Fig. 3). Concrete cisterns, as seen in community B, can harbor high fecal coliform counts (6), yet in this study, *Enterobacterales* (which includes coliforms) was not the most abundant order found. A recent environmental study of water resources found the ubiquitous presence of many bacteria belonging to the order *Enterobacterales* in source and treated water as well as in tap water provided through a piped infrastructure (42). This and our data suggest that although *E. coli* and other coliforms are being monitored at water treatment plants, other bacteria (as well as viruses) across many orders may be unaccounted for, underplaying the real risk to the water supply. What could be of great utility is studying First Nations water supplies from a quantitative microbial risk assessment (QMRA) perspective. This approach uses mathematical models to integrate factors like distribution, rate of occurrence, and concentration of a water contaminant of interest (protozoans, viruses, or bacteria) from the source water to the end user into an assessment that calculates the rate of risk of illness or infection (43, 44). Broadening the reference bacteria (and possibly including ARGs) in a QMRA may point to effective corrective measures that can be implemented to ensure an overall safer water supply in First Nations communities.

Cisterns often experience water stagnation between fillings that causes residual chlorine levels to fall thereby allowing bacterial growth (6, 28) and possibly explains why we observed very different microbial profiles between the cisterns and their matched water sources—along with cistern capacity, time water had been held in each cistern (data we were not able to obtain) and seasonal variation. While cisterns are not well represented in water distribution studies, water stagnation has been evaluated at other points along the water distribution system. In China, residential drinking water was shown to have increased levels of *Acinetobacter, Methylotenera, Pseudomonas,* and *Sphingomonas* species after only 12 hours of stagnation in municipal pipes (45). In the US, 1-week-stagnant water in residential plumbing showed increased biofilm formation and bacterial cell counts with a concomitant decrease in bacterial diversity (35). The cistern samples analyzed here contained many orders of bacteria including *Burkholderales*, *Rhizobiales*, and *Sphingomondales* (Fig. 3). Unfortunately, the opportunity for stagnation in cisterns is high as families, including the First Nations households participating in this study, persevere to conserve water so to not run out before the delivery of new water (4, 46). This lack of water movement and refreshment leads to the dissipation of the residual chlorine, bacterial regrowth, and potentially unsafe drinking water.

In this study, the two cisterns were located in geographically distinct regions in Manitoba and made from different materials. We observed that the concrete cistern had a higher abundance of *Enterobacterales*, *Rhodobacterales*, *Pseudomonadales*, *Sphingobacterales*, and *Flavobacteriales* compared to the fiberglass cistern. Cisterns in general carry a known level of contamination risk that can be due to improper maintenance or insufficient management of the supply water (47), factors and conditions that are fixable. There are very little empirical data on risk associated with cistern material (4–6, 28). It is important to expand this study to include more cisterns and locations to be able to make any clear conclusions.

### Conclusions

Previously, we demonstrated that water stored in cisterns in First Nations Communities can be highly contaminated with *E. coli* and fecal coliforms (5, 6, 28). The aim of this study was to determine if the poor quality of cistern water was due to the improper treatment of the source water at the water treatment plants, or if the cisterns were otherwise becoming contaminated; consequently, deficiencies within the water distribution and storage systems were also highlighted.

Despite access to water treatment plants, the households with cisterns in both communities B and D consumed water that contained a variety of bacteria from many different orders and showed the presence of diverse antimicrobial resistance genes [expectedly dominated by efflux pump encoding genes (8)]. Metagenomic analysis of the total resistome showed a difference in ARG abundance and diversity between the untreated source water and the cistern water, suggesting that the contamination seen in cistern water was not due to improper treatment of the source water; this was verified through PCoA.

We are aware that the restricted number of samples that we were able to collect from these remote communities due to the COVID-19 pandemic is a limitation of our study. However, this study importantly contributes to the small body of literature on overall water quality on First Nations reserves in Canada and specifically highlights that prolonged water storage in cisterns could be a major factor contributing to poor quality drinking water in First Nations households in Manitoba. This study and future work will help in the design of long-term solutions along the entire water distribution chain that will ensure the delivery of safe water to hundreds of thousands of First Nations people and end the water crisis.
